# Derivation and characterization of new cell line from intestine of turbot (*Scophthalmus maximus*)

**DOI:** 10.1007/s11626-022-00746-y

**Published:** 2023-02-21

**Authors:** Yiping Liu, Xuefeng Ge, Chao Li, Ting Xue

**Affiliations:** School of Marine Science and Engineering, Qingdao Agricultural University, Qingdao, 266109 China

**Keywords:** Cell line, Turbot, Intestine, Characterization, Gene expression

## Abstract

A continuous intestine cell line from turbot (*Scophthalmus maximus*) designated as SMI was established utilizing the tissue explant technique. Primary SMI cell was cultured at 24 °C in a medium with 20% fetal bovine serum (FBS), then subcultured in 10% FBS after 10 passages. Impacts of medium or temperature on the growth of SMI were examined and the results indicated it grew well in DMEM supplemented with 10% FBS at 24 °C. The SMI cell line was subcultured more than 60 times. Karyotyping, chromosome number, and ribosomal RNA genotyping analysis revealed that SMI had a modal diploid chromosome number of 44 and originated from turbot. After being transfected with pEGFP-N1 and FAM-siRNA, a large number of green fluorescence signals were observed in SMI, indicating that SMI could be used as an ideal platform to explore gene function in vitro. In addition, the expression of epithelium-associated genes such as *itga*6, *itgb*4, *gja*1, claudin1, *zo*-1, and E-cadherin in SMI suggested the SMI had some characteristics of epidermal cells. The upregulation of immune-associated genes such as *TNF*-*β*, *NF-κB*, and *IL*-1*β* in SMI after stimulation with pathogen-associated molecular patterns suggested the SMI might exhibit immune functions similar to the intestinal epithelium in vivo.

**Supplementary Information:**

The online version contains supplementary material available at 10.1007/s11626-022-00746-y.

## Introduction

Turbot (*Scophthalmus maximus*, *S. maximus*) is an important mariculture species in China. However, the frequent occurrence of diseases caused by bacteria and viruses seriously affected the development of turbot culture, and the possible pathological mechanisms are rarely reported.

Cell lines derived from fish provided a powerful tool for studying epidemiology (Ciotti *et al*. 2020), toxicology (Garcia *et al*. 2016), pathology (Shichi *et al*. 2022), and immunology (Faber *et al*. 2021), and for the isolation and identification of fish viruses (Pham *et al*. 2020; Li *et al*. 2022a). It also acted as a tool to study gene function of cell-derived tissues (Morin *et al*. 2020).

Since the first teleost fish cell line was isolated from rainbow trout (*Oncorhynchus mykiss*) in 1962 (Wolf and Quimby 1962), to date, more and more fish cell lines were established (Gong and Pan 2022; Li *et al*. 2022b). The establishment of teleost cell lines also can be used as in vitro models for drug screening and toxicity testing. However, to our knowledge, the cell line originating from *S. maximus* intestine has not yet been reported.

The intestinal mucosa is an important immune organ of fish. The damage of intestinal mucosa often leads to the spread of toxic and harmful factors through the blood system to other organs, which seriously affected the health and growth of the fish (Rombout *et al*. 2011). Under the stimulation of pathogens and inflammatory cytokines, immune-related pathways in intestinal tissues were activated, and antimicrobial peptides, cytokines, and chemokines were secreted to mucus and lamina propria to play a natural defense role (Rombout *et al*. 2014). Intestinal tissue, especially intestinal epithelium, not only separated the underlying tissues from potential harmful factors of the environment, but also played an important role in maintaining intestinal balance and coordinating innate and adaptive immune responses (Rombout *et al*. 2014; Parra *et al*. 2016). Therefore, the isolated turbot intestinal cells will provide models for studying the resistance mechanism of fish intestinal tract to pathogens (bacteria and viruses).

In this study, a cell line derived from the intestine of turbot (SMI) was established and characterized. The cell line had high transfection efficiency, which will provide an ideal research platform to explore the gene function. In addition, SMI showed high expression levels of epithelial cell characteristic genes and immune-associated genes.

## Materials and methods

### Primary cell culture and subculture of SMI cells

Healthy juvenile turbot about 50 g was purchased from a fish farm in Haiyang, China. The experimental protocols were approved by the Committee on the Ethics of Animal Experiments of Qingdao Agricultural University IACUC (Institutional Animal Care and Use Committee).

The isolation of turbot intestinal cell was performed as previously reported (Xue *et al*. 2018). The experimental fish was first anesthetized with MS-222 (Sigma, St. Louis, MO). The hindgut tissue was collected and washed 3 times with PBS (phosphate-buffered saline) (Cytiva, Marlborough, MA), and then soaked for 2 h in DMEM (Dulbecco’s modified Eagle’s medium) (Gibco, Grand Island, NY) containing 500 IU/mL penicillin, 500 μg/mL streptomycin, 12.5 μg/mL amphotericin B, and 250 μg/mL gentamicin (Gibco). Then the intestinal tissue was cut into small pieces (1–2 mm^3^ in size) and seeded into 25-cm^2^ flasks (Corning, Corning, NY). After the tissue was attached to the flask for 1 h, 5 mL fresh medium containing 10% FBS (Gibco), 20 nM HEPES (Gibco), 100 IU/mL penicillin, 100 μg/mL streptomycin, 55 nM β-mercaptoethanol (Gibco), and 1 × NEAA (non-essential amino acids) (Gibco) were added into 25-cm^2^ flasks. The flasks were put in the 24 °C incubator (Thermo, Waltham, MA) and two-thirds of the medium was changed every 3 d. Cells formed as monolayers were digested with 0.25% trypsin–EDTA (Gibco) solution and then transferred into a new 25-cm^2^ flask.

### Cryopreservation and resuscitation of SMI cells

The SMI cells at the exponential stage were trypsinized with 0.25% trypsin–EDTA solution, and harvested by centrifugation (300 g for 5 min) (Eppendorf, Hamburg, Germany) for cryopreservation (every fifth passage). The cells were suspended with a cold medium containing 20% FBS and 10% DMSO (dimethyl sulfoxide, MP Biomedicals, Santa Ana, CA) at a density of 5 × 10^6^ cells/mL. Cryovials containing the cells were transferred into a Biosharp Cryo Freezing Container and stored at − 80 °C overnight, and then transferred to liquid nitrogen for long-term storage. After storage for 1 mo, one cryovial containing SMI cells was taken from liquid nitrogen and placed in a water bath (37 °C). The cryovial was shaken quickly to thaw the cryopreservation solution within 1 min. The cells were transferred to a 15-mL centrifuge tube containing 9 mL fresh medium. SMI cells were collected and transferred to a 25-cm^2^ flask and cultured at 24 °C. After 12 h, photographs were taken to record cell status and the medium was replaced with fresh medium.

### Optimization of SMI culture conditions

The growth rate of the 52^nd^ passage SMI at different temperature, culture medium, or FBS concentration was assessed as described previously (Xue *et al*. 2018). To test the optimal culture temperature, the cells were incubated at different temperatures of 16 °C, 20 °C, 24 °C, 28 °C, and 32 °C for 5 d. Analogous procedures were performed to detect the effects of different mediums (L-15 (Leibovitz’s L-15) (Cytiva), DMEM, DMEM: F12 (Dulbecco’s Modified Eagle’s Medium/Ham’s F-12) (Gibco), RPMI 1640 (Roswell Park Memorial Institute 1640) (Gibco), and M199 (Medium 199) (Gibco), contained 10% FBS) and concentrations of FBS (2, 5, 10, 15, and 20%, in DMEM) to SMI growth at 24 °C. In order to test the optimum conditions of temperature, basal medium, and serum concentration for SMI growth, SMI cells were seeded in 48-well plates at a density of 4 × 10^4^ cells per well. After 24 h of inoculation (all cells adhered to the plate), replaced the old medium with the medium that test the best basic medium and serum concentration and cultured the cells in different conditions. Then, 3 wells SMI cells of various mediums were trypsinized daily and counted 3 times using a hemocytometer. The average number of cells at various conditions was used in constructing a growth curve. The experiments were conducted in triplicate.

### The origin of SMI cells

The karyotype was analyzed using SMI cells at the 10^th^ passage. The cells at the exponential stage were treated with 10 µg/mL colchicine (Solarbio, Beijing, China) for 12–16 h at 24 °C. The cells were trypsinized with 0.25% trypsin–EDTA and harvested. Fixation and staining were performed as previously reported (Xue *et al*. 2018). The number of chromosomes was counted in one hundred metaphase cells under a ZEISS Axio Scope A1 microscope (ZEISS, Jena, Germany), and chromosome karyotype was analyzed according to a previous publication (Levan *et al*. 1964).

The species origin of the SMI cell line was validated by amplification and sequencing of 18S rRNA (*XR_004790016.2*) using primers 18SF and 18SR. Genomic DNA of the 50^th^ passage SMI was extracted using DNA extraction kit (TIANGEN, Beijing, China) according to the instructions. The PCR product was prepared for sequencing. The sequence was aligned against the known *S. maximus* 18S rRNA sequence by using DNAMAN software.

### Transient transfection of SMI cells for plasmid and siRNA

The SMI cells from passage 53 were selected to test its transient transfection efficiency for plasmid and siRNA. The SMI cells were seeded in a 6-well plate (at a density of 5 × 10^5^ cells/well). About 60% confluent monolayers were transfected with 5 μg pEGFP-N1 plasmid or 100 pmol FAM-siRNA (GenePharma, Shanghai, China) using Xfect™ Transfection Reagent accordingly manually (Takara, Kusatsu, Japan). The proportion of fluorescence-positive cells was recorded under ZEISS Axio Observer 7 fluorescence microscope (ZEISS) after 48-h transfection.

### Specific gene expression of SMI cells after stimulation with LPS or poly (I:C)

Expression of two gene sets, immune-related genes and epithelial-related genes, was studied in control SMI and SMI exposed to either LPS or poly (I:C). The immune-related genes were *TNF*-*β* (tumor necrosis factor β), *NF-κB* (nuclear factor-κB), and *IL-*1*β* (interleukin-1β). The epithelial-related genes were *itga*6 (integrin α6), *itgb*4, *gja*1 (gap junction α1, also named connexin 43), and claudin1. SMI cells from passage 55 were seeded into 12-well plates with 1 × 10^5^ cells/well. After the cell growth and confluency reached 80%, they were stimulated with 20 μg/mL LPS (lipopolysaccharide) (Solarbio) or 20 μg/mL poly (I:C) (polyinosinic-polycytidylic acid) (APExBIO, Houston, TX). The control group was treated with an equal volume of PBS. Three replicates were set for each treatment condition. Cells were collected at 2, 12, and 24 h after stimulation for RNA extraction.

### The culture of SMI in Transwell-system

SMI cells from passage 55 were seeded at a density of 5 × 10^5^ cells/well in a 6-well plate. The cells (as control) were harvested after growth and confluency reached 80% for RNA extraction. SMI cells from passage 55 were seeded at a density of 6 × 10^4^ cells/well on three polyester filter Transwell inserts (LABSELECT, Hefei, China, 0.4-μm pore size, 24-mm surface diameter). Medium was changed after the first 3 d and then every other day for all experiments. The SMI cells grown on Transwell inserts for 21 d were collected for RNA extraction. The expression of *itga*6, *itgb*4, *gja*1, claudin1, *zo*-1*a* (zonula occludens 1a), *zo*-1*b*, and E-cadherin were detected in control and Transwell-system SMI cells.

### RT-qPCR (real-time fluorescent quantitative PCR)

The total RNA was extracted using TRIzol® Reagent (Invitrogen, Waltham, MA) according to the supplied protocol. The cDNA was synthesised using PrimeScript RT Reagent Kit (Takara). RT-qPCR was performed on CFX96 real-time PCR detection system (Bio-Rad Laboratories, Hercules, CA) using TB Green Premix Ex Taq™ qRT-PCR Kit (Takara). The RT-qPCR was repeated three times for each sample. The expression level of genes was calculated using the comparative Ct method (2^−ΔΔCt^) (Livak and Schmittgen 2001). The primers are shown in Table 1. The 18S rRNA (18SqF and 18SqR) was used as an internal control. The amplification efficiency of all the primers was detected, and the results were presented in Supplementary Fig. 1. The fold change expression levels of different genes were visualized using GraphPad Prism 7.

**Table 1.** Primers used in this study

Primer name	Nucleotide sequence (5′-3′)	Purpose	Accession number
18SqF	TGTGGGTTTTCTCTCTCTG	**RT-qPCR**	***XR_004790016.2***
18SqR	ATTCTTGGCAAATGCTTTC		
18SF	CGCTTTGGTGACTCTAGATAACCT	**Fragments amplification**	***XR_004790016.2***
18SR	CCATTATTCCTAGCTGCGGTATT		
IL-1βqF	GAGGCAGTGACAACCGCAAAG	**RT-qPCR**	***XM_035640817.2***
IL-1βqR	TTGGCACGACAGGTAGAGATT		
TNF-βqF	CCAAGGCAGCCATCCATTTAG	**RT-qPCR**	***XM_035629860.2***
TNF-βqR	ATGATCTGGTTCTCCATCAGC		
NF-κBqF	GCGTTGCTGCACAACTCATCC	**RT-qPCR**	***XM_035650517.2***
NF-κBqR	TCTGTCTGGGAAGGCTGGAAG		
itga6qF	GGTGACGGTTCAGAGCCAGG	**RT-qPCR**	***XM_035604681.2***
itga6qR	TAGCAGCGCCCAGTGATGTC		
itgb4qF	TATGTGGTCACCTGCGAACAG	**RT-qPCR**	***XM_035612571.2***
itgb4qR	GGACTTTGAACTTGTAGGGAACG		
zo-1aqF	TCAGGTGGACGAGACAACCCT	**RT-qPCR**	***XM_035627666.2***
zo-1aqR	ACAGCATTGACCATGACCACT		
zo-1bqF	ATGGATAATGTGGAGCATGCG	**RT-qPCR**	***XM_047333104.1***
zo-1bqR	ACCTTCCTCTTCCGTCTGATT		
E-cadherinqF	GGGCATCATCATAGGCAACAC	**RT-qPCR**	***MG137250.1***
E-cadherinqR	CTTCTTGGGTTGACCATCGTT		
claudin1qF	CTGGGCTGGTCCCTATACCT	**RT-qPCR**	***MT345531.1***
claudin1qR	GGGTGTAGTTCTTGCGGTGA		
gja1qF	TGGTCCTGGGTACCGCAGTG	**RT-qPCR**	***KY677742.1***
gja1qR	AGGACTTGTCATAGCAGACGTT		

### Statistical analysis

Each experiment was repeated at least three times and expressed as mean ± SD. The data were analyzed by one-way ANOVA followed by a post hoc Tukey’s test using SPSS 20.0 (SPSS, Chicago, IL), and the *p* ≤ 0.05 denoted a statistically significant difference.

## Results

### Primary culture and subculture

**Figure 1.**
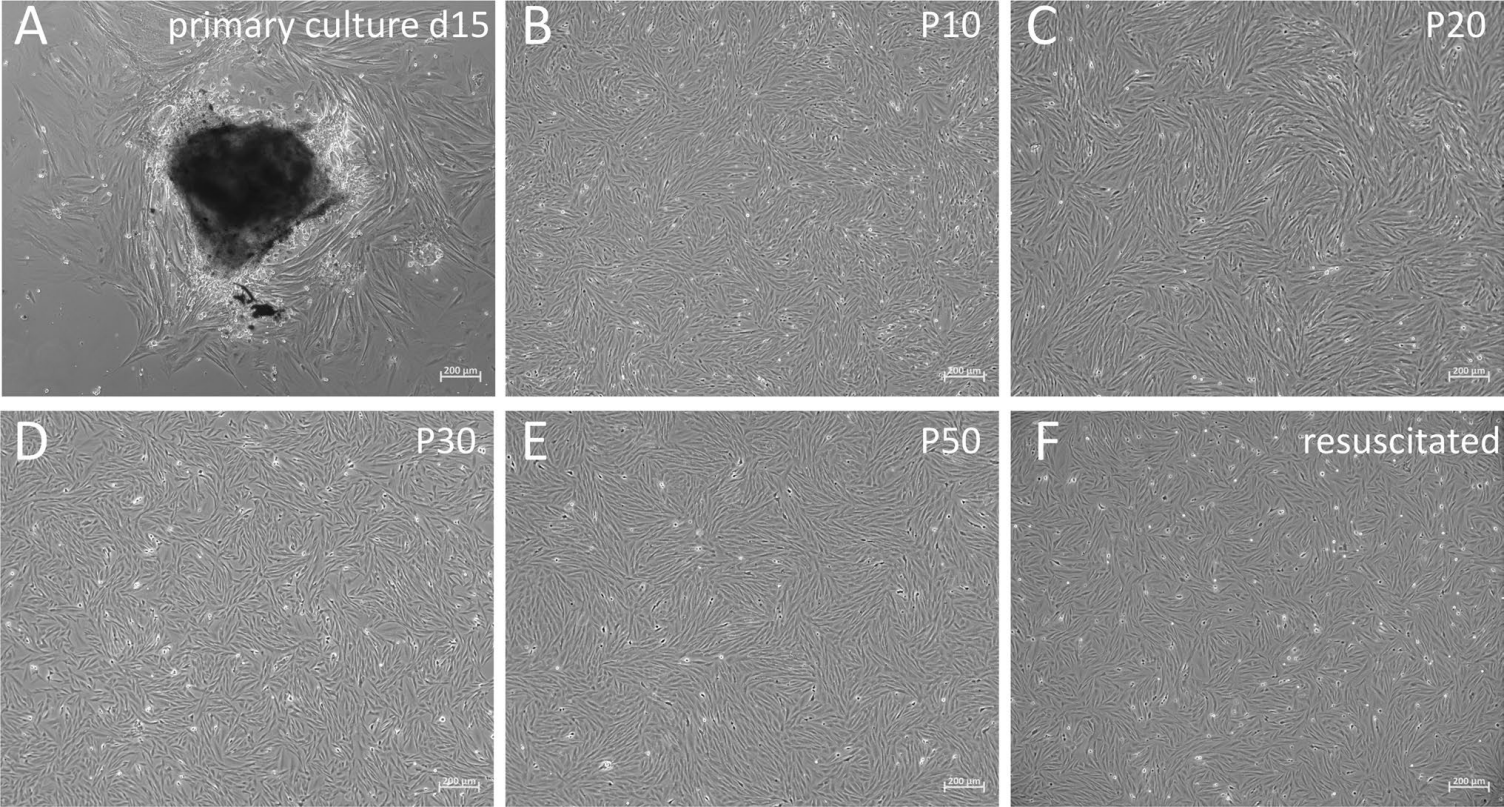
Photomicrography of primary cultured, subcultured, and resuscitated intestine cells derived from turbot. *A* Primary cells cultured for 15 days; *B* passage 10 cells; *C* passage 20 cells; *D* passage 30 cells; *E* passage 50 cells; *F* resuscitated SMI.

The SMI cells migrated from the edge of the *S. maximus* hindgut tissue after 15 d of primary culture (Fig. 1*A*). The cells reached 80% confluence in 25-cm^2^ flasks on the 25^th^ day and then subcultured every 4–7 d (ratio of 1:2). After 10 passages, the FBS concentration was gradually decreased from 20 to 10%, and cells were subcultured at a ratio of 1:3. The SMI was mainly composed of fibroblast-like cells (Fig. 1*B*–*E*). SMI has been subcultured more than 60 passages. The SMI cells cryopreserved at different passages all showed > 90% viability after storage in liquid nitrogen for 1 mo and grew to confluence within 3 d. There was no obvious change in morphology and growth rate after freezing and thawing (Fig. 1*F*).

### Characterization of cell growth

**Figure 2.**
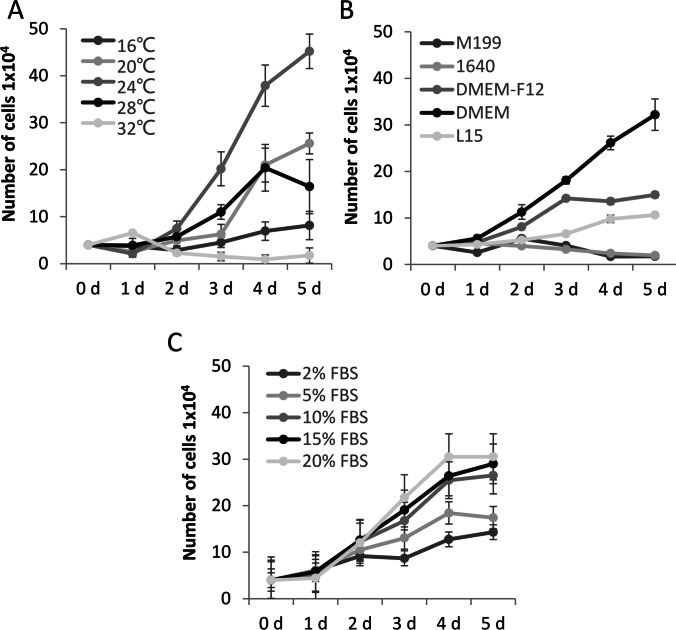
The growth curve of SMI cells in different incubation temperatures (*A*), different basal mediums (*B*), and different concentrations of fetal bovine serum (*C*). Data are shown as mean ± SD of three measurements.

To optimize the culture condition, the growth rate of SMI at 52^nd^ passage under different conditions was investigated. A temperature range of 16–32 °C was used to test the optimal growth temperature (Fig. 2*A*). L-15, DMEM, DMEM: F12, RPMI 1640, and M199 were used to test the optimal basal culture medium (Fig. 2*B*). The FBS concentration range of 2–20% was used to test the optimal FBS concentration (Fig. 2*C*). The results showed that the optimum temperature was at 24 °C (Fig. 2*A*), the SMI proliferated rapidly in DMEM and DMEM: F12 medium (Fig. 2*B*), the growth rate of SMI cells increased as the FBS concentration increased from 2 to 20% (Fig. 2*C*). To minimize the cost, DMEM + 10% FBS and cultured at 24℃ was selected as the optimal culture conditions for SMI.

### The origin of the SMI

**Figure 3.**
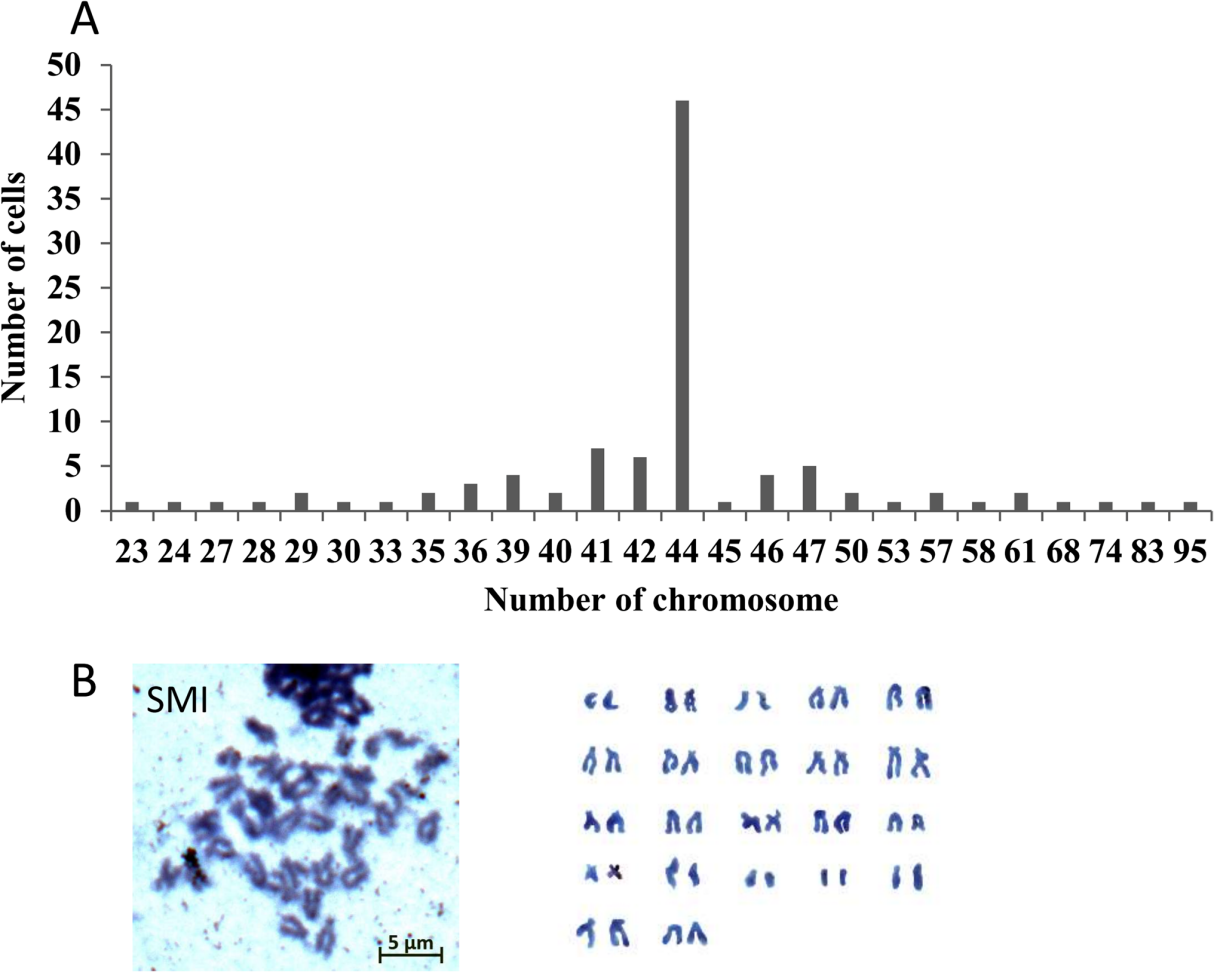
Frequency distribution of chromosome number (*A*), and chromosome metaphase and karyotype (*B*) for 10 passage SMI cells

The analysis of the metaphases of 100 number of 10^th^ passage SMI cells revealed that 46% of the cells had 44 chromosomes (Fig. 3*A*). Meanwhile, heteroploidy (chromosome numbers varied from 23 to 95) was observed in the SMI cell line as a small proportion (Fig. 3*A*). The metaphase with a normal diploid number of 44 displayed the normal karyotype morphology (Fig. 3*B*).

**Figure 4.**
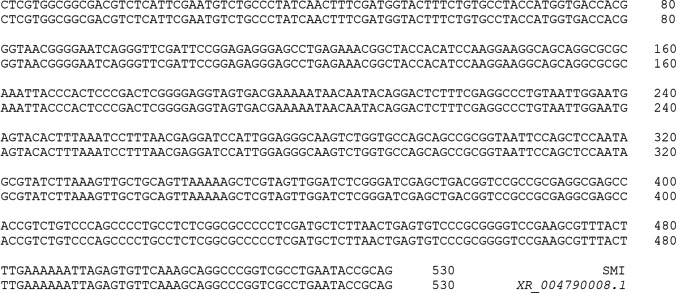
The alignment of 18S rRNA sequence of turbot SMI with part NCBI sequence of *XR_004790016.2* (https://www.ncbi.nlm.nih.gov/).

The species origin of the cell line was verified using 18S rRNA. A 530-bp fragment of 18S rRNA was amplified in SMI. The sequencing result indicated that the 530-bp fragment showed 100% identity with published turbot 18S rRNA (*XR_004790016.2*) (Fig. 4).

### Transient transgenic analysis

**Figure 5.**
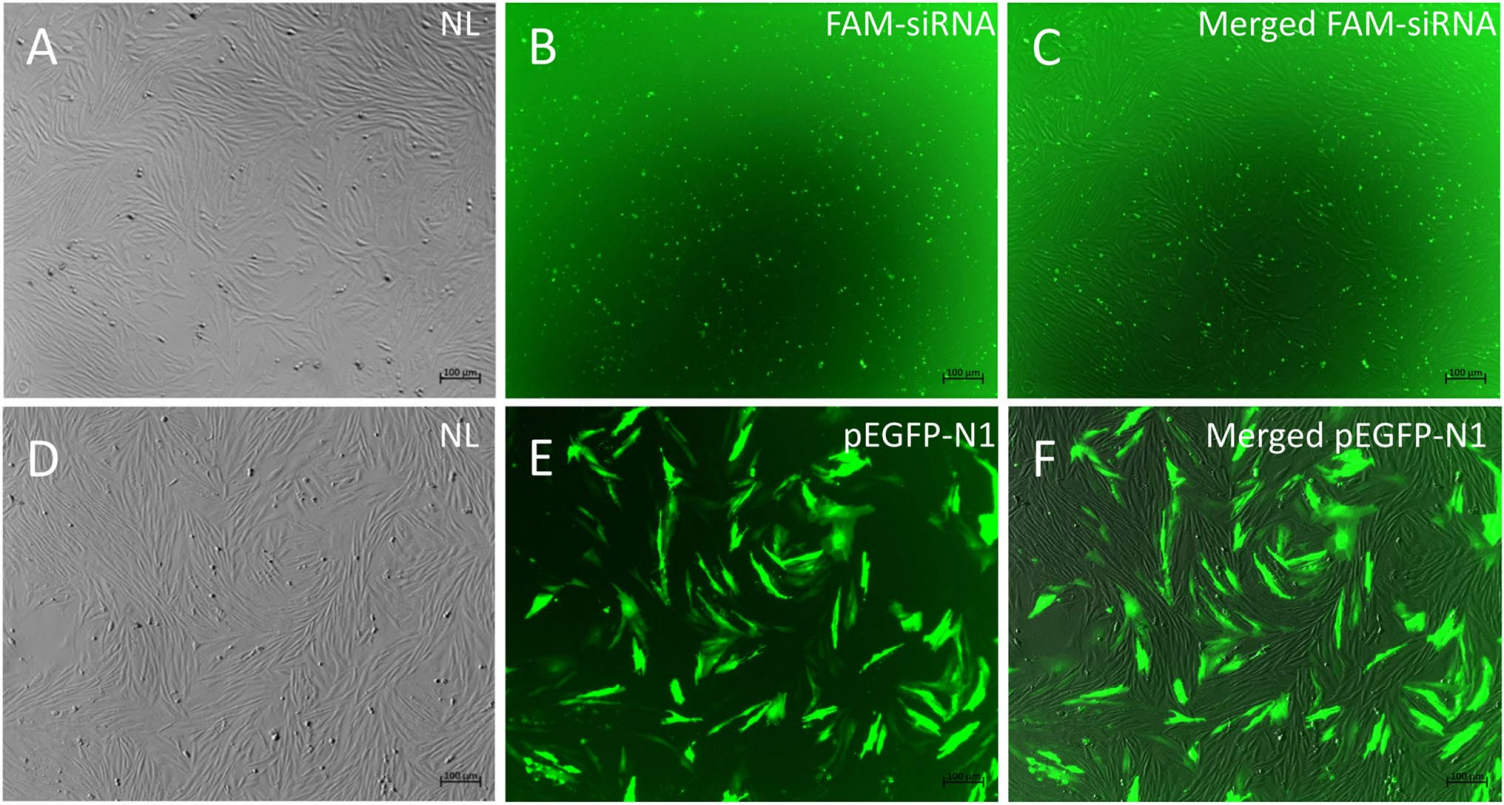
The detection of fluorescent signals in SMI at passage 53 after transfection. SMI cells were transfected with FAM-siRNA or pEGFP-N1 plasmid, and the fluorescence signals were observed under fluorescence microscope. (*A*–*C*) SMI cells transfected with FAM-siRNA; (*D*–*F*) SMI cells transfected with pEGFP-N1. NL, nature light. *Scale bars*, 100 μm.

The SMI cells were successfully transfected with pEGFP-N1 plasmid or FAM-siRNA using Xfect™ Transfection Reagent. Strong green fluorescence signals of EGFP protein (Fig. 5*A*–*C*) or FAM were observed SMI cells at 48 h after transfection (Fig. 5*D*–*F*). It indicated the suitability of this cell line for gene overexpression and interference studies.

### Gene expression

**Figure 6.**
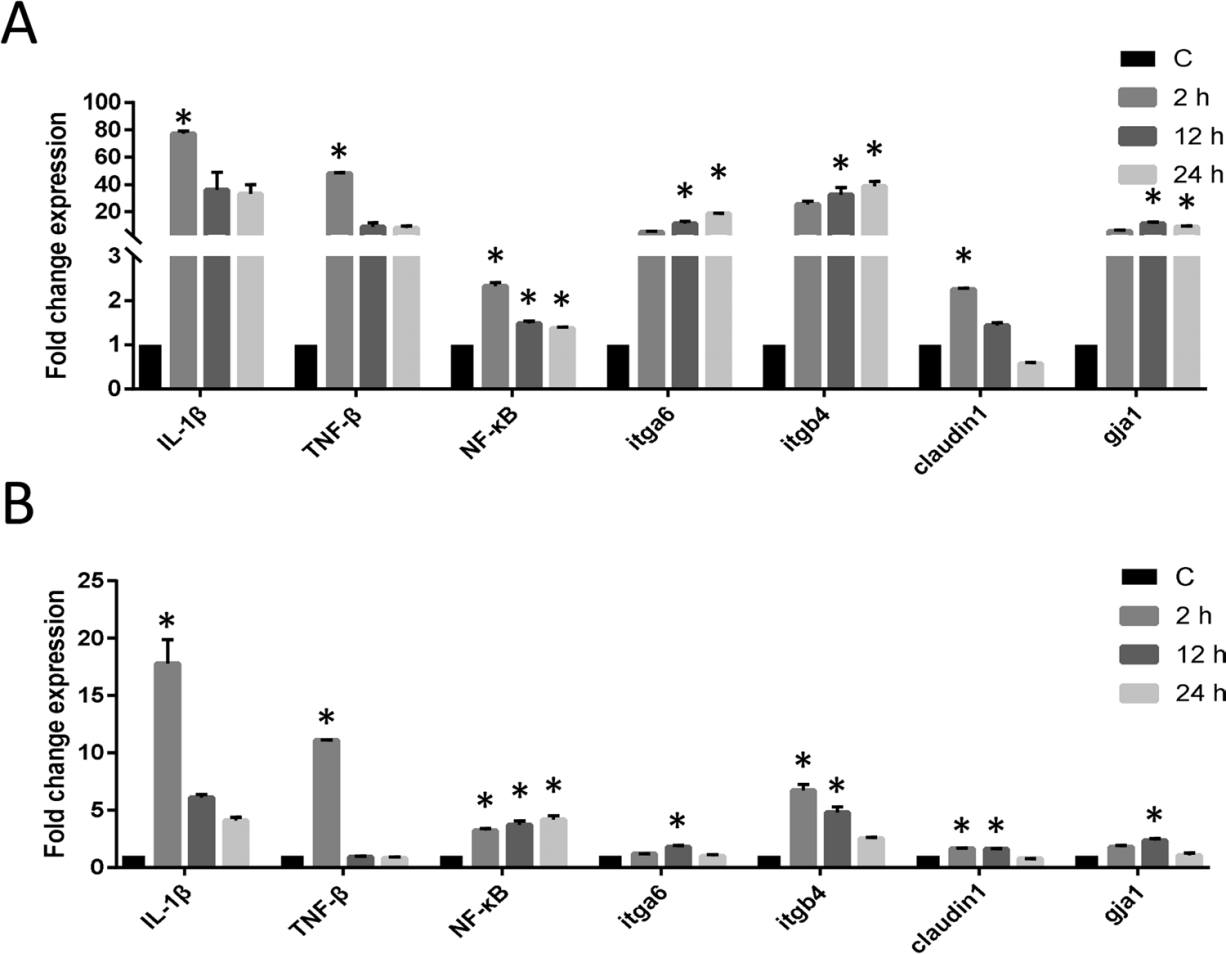
The expression levels of immune-associated genes and epithelium-associated genes in control SMI and the SMI after stimulation with LPS (*A*) and poly (I:C) (*B*) for 2 h, 12 h, and 24 h. *C* Control group. *p ≤ 0.05.

The expression of immune-related genes *TNF*-*β*, *NF-κB*, and *IL-*1*β*, and epithelial-related genes such as *itga*6, *itgb*4, *gja*1, and claudin1 were analyzed at the 55^th^ SMI cell line. All of those genes were strongly expressed in the SMI cells. The expression levels of *IL*-1*β* and *TNF*-*β* were upregulated at the early stage after the stimulation of both LPS and poly (I:C), and *NF-κB* was upregulated at all the time points after being stimulated with LPS and poly (I:C) (Fig. 6*A*, *B*). The expression levels of *itga*6, *itgb*4, *gja*1, and claudin1 were all upregulated at all the time points after being stimulated with LPS (Fig. 6*A*). After being stimulated with the poly (I:C), these genes were also upregulated at the early stage, but returned to normal in the later stages (Fig. 6*B*).

**Figure 7.**
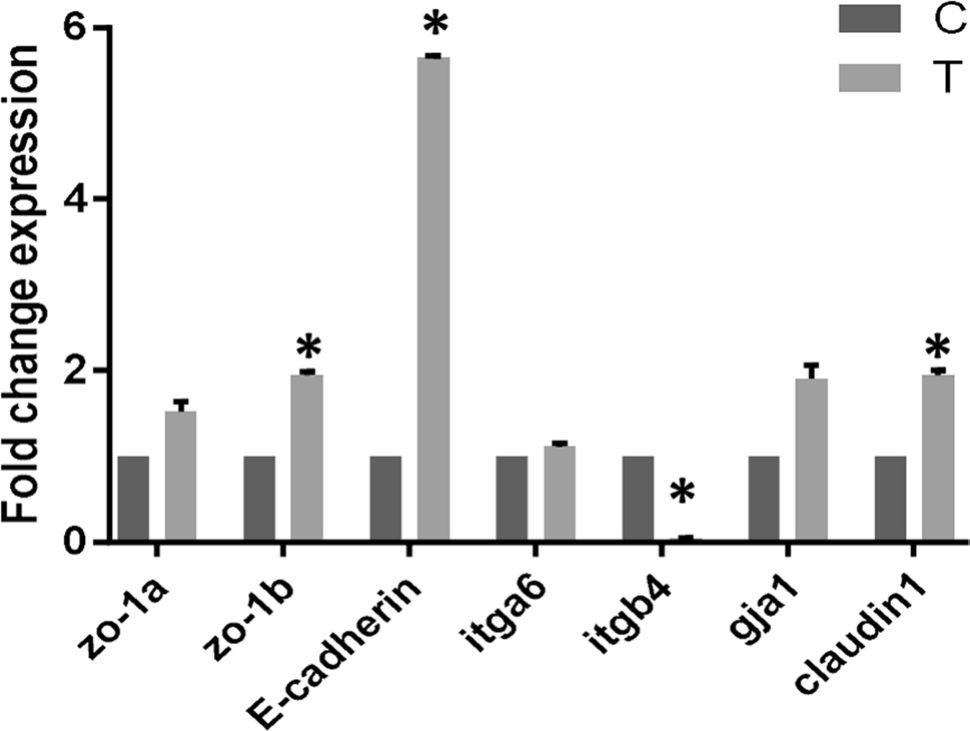
The expression levels of *itga*6, *itgb*4, *gja*1, claudin1, *zo*-1*a*, *zo*-1*b*, and E-cadherin in SMI that seeded in normal culture plates or Transwell-system. C, normal culture plates; T, Transwell-system. *p ≤ 0.05.

The expression levels of epithelial-related genes *itga*6, *itgb*4, *gja*1, claudin1, *zo*-1*a*, *zo*-1*b*, and E-cadherin in the SMI cells that seeded in the normal culture plates and Transwell-system were compared. The results showed that most of the detected genes were upregulated in the SMI cells seeded into the Transwell-system relative to normal cultured SMI cells (Fig. 7). Among those genes, the expression level of claudin1, *zo*-1*b*, and E-cadherin was significantly upregulated. However, the expression level of *itgb*4 was downregulated in the Transwell-system.

## Discussion

*S. maximus* is a commercially important freshwater fish species that is widely cultured in the north of China (Xiao *et al*. 2008). However, the diseases caused by intestinal pathogenic bacteria are seriously affecting the development of turbot aquaculture (Olsson *et al*. 1998; Gao *et al*. 2021). It is of great significance to study the infection mechanism of these pathogenic bacteria. Relative to the individual, the cell line has a higher experimental repetition rate. Therefore, a new cell line (SMI) derived from the intestinal tissue of *S. maximus* was established to study the pathogenic mechanism of intestinal pathogens. The cell line was established by means of the explant technique. It is the same as the establishment of the liver (Li *et al*. 2022b), fin (Yashwanth *et al*. 2020), brain (Ruiz-Palacios *et al*. 2020), skin (Vo *et al*. 2019), kidney (Liu *et al*. 2022), and gill (Sathiyanarayanan *et al*. 2022) tissue in other teleosts.

DMEM supplemented with 20% FBS at 24 °C was identified as the optimal condition for the growth of SMI cells, similar to other turbot cell lines (Fan *et al*. 2010; Gao *et al*. 2019; Wang *et al*. 2010). To date, the SMI cell line has been subcultured for more than 60 passages. The morphology of SMI cells remained consistently fibroblast-like from their initial to the current passage. By contrast, cultures of the rainbow trout intestinal cell line (RTgutGC) became more epithelial-like with passaging (Kawano *et al*. 2011). Karyotype analysis revealed that 46% of the SMI cells possessed a diploid chromosome number of 2n = 44, which was identical to the modal number of other turbot cell lines such as TMF (Gao *et al*. 2019), TF (Fan *et al*. 2010), and TK (Wang *et al*. 2010). 18S rRNA has frequently been used in species identification and classification among aquatic and mammalian species (Liu *et al*. 2021; Zheng and Yang 2020). The sequencing of 18S rRNA indicated that the SMI cells were derived from turbot. To validate the suitability of SMI as an in vitro tool to study the function of intestine-specific genes, the transient transfection efficiency of SMI cells for plasmid and siRNA was detected. The results demonstrated that the transfection efficiency of SMI cells for plasmid and siRNA was higher than that in some other fish cell lines used for immune-related studies, such as EPC and RTG2 (Falco *et al*. 2009).

*TNF*-*β*, *NF-κB*, and *IL-*1*β* are classical immune-related genes that are highly expressed in different immune cells of many species (Zhang *et al*. 2021). After *Vibrio alginolyticus* (*V. alginolyticus*) infection, intraperitoneal injection of recombinant *IL*-1*β* improved the survival rate of large yellow croaker (*Larimichthys crocea*) and reduced the tissue bacterial load. Simultaneously, recombinant *IL*-1*β* reduced bacterial killing capability in ayu (*Plecoglossus altivelis*) head kidney–derived monocytes/macrophages (Lu *et al*. 2013). The mRNA levels of *TNF*-*β* in Nile tilapia (*Oreochromis niloticus*) spleen lymphocytes were significantly upregulated during the adaptive immune stage after *Streptococcus agalactiae* infection (Li *et al*. 2021). The *NF-κB* signaling system plays an important regulatory role in the control of pathophysiological situations such as inflammation and infection (Meier-Soelch *et al*. 2021; Wei *et al*. 2021). The expression of *TNF*-*β*, *NF-κB*, and *IL-*1*β* genes in SMI cells suggested that SMI might be used as an in vitro model for the study of intestinal immune function. The upregulation of *TNF*-*β*, *NF-κB*, and *IL-*1*β* in SMI after LPS and poly (I:C) stimulation indicated that pathogen-associated molecular patterns activated the defense of SMI.

*Itga*6, *itgb*4, *gja*1, and claudin1 genes are usually expressed in the epithelial cells. Integrin α6β4 was one of the main laminin receptors and was primarily expressed by epithelial cells that line the luminal surface of the colonic crypts (Beaulieu 2019). *Gja*1 has been thought to be involved in the pathophysiology of a variety of intestinal epithelial (IEC) barrier diseases, including inflammatory bowel diseases, necrotizing enterocolitis, and enteric infection (Dubina *et al*. 2002; Velasquez Almonacid *et al*. 2009). In acute inflammatory stress–induced damage, TLR2-mediated mucosal healing was functionally dependent on intestinal epithelial *gja*1 (Ey *et al*. 2009). Claudin-1 was specifically expressed in mouse epithelial cells. It could regulate intestinal epithelial homeostasis by modulating the Notch-signaling pathway, and its overexpression could alter the differentiation of intestinal epithelial cells (Pope *et al*. 2014). The upregulation of *itga*6, *itgb*4, *gja*1, and claudin1 in SMI after LPS and poly (I:C) stimulation indicated that SMI might play part of the function of turbot intestinal epithelial cells.

As members of the tight junction family, *zo*-1 and claudins play an essential role in intestinal epithelial intercellular junctions. Expression of the *zo*-1 was confirmed in rainbow trout intestinal epithelial cell line by immunofluorescence (Pumputis *et al*. 2018). E-cadherin and claudin can interact with *zo*-1 to maintain the intestinal epithelial barrier and signal transduction between adjacent cells, and they were also expressed in the rainbow trout intestinal cells (RTpi-MI and RTdi-MI) that can form the intestinal epithelial barrier (Pasquariello *et al*. 2021). The upregulation of *itga*6, *itgb*4, *gja*1, claudin1, *zo*-1, and E-cadherin in SMI cells cultured in the Transwell-system might suggest that SMI cells formed a functional barrier in the Transwell-system as other fish intestinal epithelial cell lines. Whether SMI can function as an intestinal immune barrier needs to be further studied.

In summary, a new turbot cell line SMI was established and identified. SMI cells had high transfection efficiency for both plasmid and siRNA, which will provide a possibility for functional studies of intestinal-specific genes in the future. The expression changes of immune- and epithelial-related genes in SMI cells after pathogen-associated molecular pattern stimulation also suggested that SMI cells might be used to study part of the function of intestinal mucosal immunity in vitro.

## Supplementary Information

Below is the link to the electronic supplementary material.

Supplementary file 1 (DOCX 838 KB)
