# Supplementary material for: Derivation and characterization of new cell line from intestine of turbot (Scophthalmus maximus)
Source: In Vitro Cell Dev Biol Anim. 2023 Feb 21;59(2):153–62. doi: 10.1007/s11626-022-00746-y (PMC10073165; doi:10.1007/s11626-022-00746-y)
Supplement: Supplementary file 1 — (DOCX 838 KB) [file 11626_2022_746_MOESM1_ESM.docx]

Supplementary Figure 1 Assessment of primer efficiency. The efficiency of all the primers pair used in qRT-PCR was evaluated by plotting the cycle threshold value (Ct) at each dilution against the logarithm of the fold dilution of the sample. The efficiency of the primers is calculated from the slope of the standard curve.


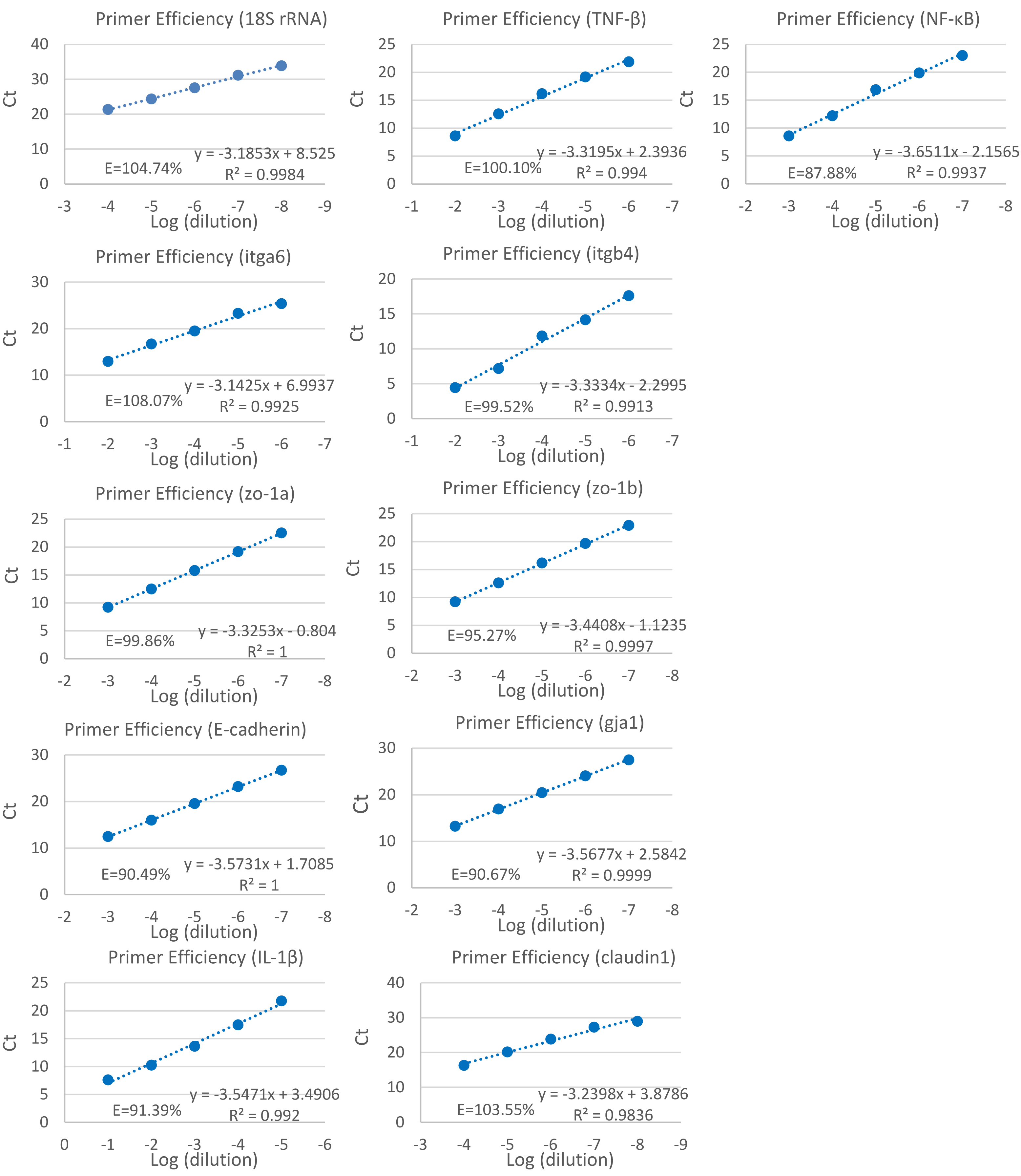


The evaluation method of primer efficiency is shown as follows:

The efficiency of all the primers pair used in qRT-PCR was evaluated. The amplification efficiency of the primers was determined as follows: prepare a dilution series of a cDNA template (eight 10-fold dilutions from an initial concentration of ~10 ng) for all relevant primer pairs and titrate the in duplicate parallel reactions; construct standard curves for the target by plotting Ct values (Y-axis) against the log of template amount or dilution (X-axis); calculate primer efficiency by using the formula: (10(−1/slope)-1) * 100.
